# Motivators and barriers for smoking cessation in people with multiple sclerosis: a qualitative study to inform the design of a tailored intervention

**DOI:** 10.1186/s12889-024-20998-5

**Published:** 2024-12-17

**Authors:** Alex M. Keller, Barbara von Glasenapp, Daniel Kotz, Claudia H. Marck, Christoph Heesen, Karin Riemann-Lorenz

**Affiliations:** 1 Institute of Neuroimmunology and Multiple Sclerosis (INIMS), University Medical Centre Hamburg- Eppendorf, Martinistrasse 52, Hamburg, 20246 Germany; 2 Institute of General Practice, Addiction Research and Clinical Epidemiology Unit, Centre for Health and Society, Medical Faculty and University Hospital Düsseldorf, Heinrich Heine University Düsseldorf, Düsseldorf, Germany; 3 Disability and Health Unit, Centre for Health Policy, the Melbourne School of Population and Global Health, The University of Melbourne, Melbourne, VIC Australia

**Keywords:** Multiple sclerosis, Smoking cessation, Qualitative study, Intervention development, Health behaviour

## Abstract

**Background:**

Tobacco smoking is a relevant determinant of multiple sclerosis (MS) onset, and smokers have increased risk for faster progression of MS compared to non-smokers. While the smoking prevalence is high in Germany, no smoking cessation programs have been developed specifically in MS populations to date, and only little is known about the motivators and barriers influencing smoking cessation in people with MS (pwMS) in Germany. This study aims to identify these factors to inform the design of a tailored smoking cessation intervention.

**Methods:**

As part of a larger program of work, we conducted semi-structured interviews in people with MS (pwMS) to explore their needs, motivators and barriers regarding smoking cessation. We recruited via MS-websites and the email-newsletter of our institution at the University Medical Centre Hamburg-Eppendorf. Participants were eligible if they had a self-reported MS-diagnosis and currently smoked or quit smoking within the last two years but after their MS-diagnosis. Interviews were conducted online and via telephone during May and June 2023. Data were analysed using thematic analysis based on a realistic approach.

**Results:**

Eight women and seven men participated in our interviews. Eleven were current, four were former smokers. Median time since diagnosis was 4 years (range: 1–26). Interviews identified MS-diagnosis, concerns about general health, and social factors as relevant motivators to stop smoking. Furthermore, worries about negative consequences when quitting (e.g. fear of missing out on social interactions or weight gain) were identified as a great barrier to smoking cessation. Knowledge about the connection between MS and smoking, and satisfaction with communication with MS clinicians were low. PwMS expressed a need for better conversations with neurologists and expert-led smoking cessation interventions. Additionally, we found that the wish for peer-exchange and the willingness to participate in smoking cessation programs was high.

**Conclusion:**

Our results confirm findings of previous studies from other countries, identifying lack of knowledge, unsatisfactory communication with MS clinicians, and worries about negative consequences when quitting as barriers, and the MS-diagnosis as a motivator for smoking cessation. In a next step, we will use our findings for the development of an MS-specific online smoking cessation program.

**Supplementary Information:**

The online version contains supplementary material available at 10.1186/s12889-024-20998-5.

## Introduction

Multiple sclerosis (MS) is a chronic neurological condition, which manifests based on genetic vulnerability coupled with environmental factors. It leads to substantial disability in at least two thirds of people with MS (pwMS) [1]. According to the latest data from the German Society of Neurology, there are currently 280,000 pwMS in Germany [2]. While there is no cure for the disease, there is a wide range of disease-modifying drugs, as well as health behaviour interventions for diet and exercise available, reducing disease activity and slowing progression [3–5]. Smoking is already an established risk factor in MS-research. Contemporary studies have shown that both, smoking tobacco as well as passively inhaling tobacco smoke are associated with an increased risk of developing MS [6–9]. Smoking also accelerates disease progression compared to non-smokers with MS [10], and increases the relapse rate and mortality [8, 11, 12]. Smoking is also associated with lower quality of life [13], increased depression and anxiety [14, 15], and worse cognitive function [16] in pwMS. Additionally, there is evidence that smoking can reduce the effectiveness of MS-medication [17–19]. On the other hand, a cross-sectional study [20] showed, that smoking cessation significantly reduces the risk of reaching disability milestones. In this context, a further study [21] showed that several disease outcomes (e.g. physical disability, quality of life, cognitive performance) for pwMS did not significantly differ between never smokers and those who quit smoking after diagnosis, pointing at the positive effects of smoking cessation for pwMS [6]. As of July 2024, the prevalence of the tobacco-smoking German population aged 14 and above lies at 28.2% [22], while some German studies analysing MS-populations found prevalence of current smokers between 19% and 24% [23, 24]. But there are some studies outside Germany reporting even higher prevalences of pwMS who smoke, reaching from 38% [25] up to 49% [26]. However, a systematic review from 2017 [10] could not identify a single intervention study focusing on smoking in MS, and to our knowledge no interventions have been developed since then.

Several researchers have been calling out the lack of research in this area, demanding more research on the topic of smoking cessation in pwMS [27, 28]. They argue, that pwMS need tailored interventions, to accommodate for MS-specific motivators to continue smoking, and for MS-specific barriers preventing smoking cessation. An Australian qualitative study interviewed 25 pwMS and found, that these barriers and motivators could be e.g. the lack of knowledge about the harmful influence of smoking on MS among pwMS, and the fact that smoking was perceived by some participants as beneficial and by some as disadvantageous regarding their MS-symptoms. They conclude that, in order to meet the specific needs and wishes of pwMS, a tailored and targeted behavioural intervention is a promising step towards successful smoking cessation in contrast to generic smoking cessation programs [29]. Data on these issues about the German MS population is lacking. We expect reports about perceived benefits or disadvantages to be mainly similar compared to the Australian study. But social aspects influencing smoking behaviour could be more pronounced in German pwMS, since smoking is much more common in Germany (prevalence of 28.2% [22]) compared to Australia (prevalence of 8.3% [30]).

The main aim of this study is to identify the motivators, barriers, needs and wishes regarding smoking cessation for pwMS in Germany to inform the design of a tailored smoking cessation intervention. Hence, this study’s’ research questions are (1) What are the MS-specific motivators for tobacco smoking and barriers for smoking cessation for pwMS, and (2) What is the level of awareness about the connection between smoking and MS and what are the needs in terms of assistance for smoking cessation in pwMS?

As this study will contribute to the development of a targeted smoking cessation program for pwMS, we want to consider our findings using a theoretical framework that can explain how the interaction between barriers, motivators, knowledge and wishes can result in a behaviour change [31]. The COM-B model provides us with such a framework. According to this model ***C****apability*, ***O****pportunity* and ***M****otivation* are the driving factors for any ***B****ehaviour* to occur. Therefore, it can be used to purposefully identify what needs to change for a new behaviour to arise [31]. In the discussion, we will give an overview, on how our results could fit onto this model.

## Methods

The report of methods and findings used in this study adheres to the consolidated criteria for reporting qualitative studies (COREQ) [32]. The filled-out checklist can be found in our supplementary materials.

### Research team

Our interdisciplinary research team comprised six people from the fields of neurology, health sciences, physiotherapy, psychology and public health. The interviewer (AMK) has a background in health sciences and works as a research assistant. He had previous experience in conducting and analysing interviews from unrelated projects. No relationship with the participants was established prior to study commencement and only the occupation of the interviewer was known to the participants.

### Participant selection

Smoking cessation is a heavily under-researched topic in MS care [28]. Hence, we aimed to include a broad range of perspectives in our data by applying a purposive sampling strategy, aiming to recruit around 15 to 20 participants with a good variety within our sampling criteria, which were: sex, different types of MS (relapsing remitting, primary progressive and secondary progressive) and different stages of disability. Disability was measured with the Patient Determined Disability Scale (PDDS), a simple and validated ordinally scaled tool for self-assessment of disability that ranges from 0 “normal functionality” to 8 “bedridden” [33]. Participants were approached via an email-newsletter of our department at the University Medical Centre Hamburg-Eppendorf, or with posters, flyers and direct referrals from MS clinicians in our and a partnered facility (Marianne-Strauss-Clinic, Berg, Germany), and via the website of the German Society for MS (DMSG). We recruited 16 participants in the period from May to July 2023 and stopped when it was agreed that data saturation was reached. No potential participants had to be turned down. One dropped out without specifying a reason. Hence, interviews were conducted with 15 participants. Participants received no payment for participation.

### Setting

To facilitate the participation of our participants, we offered to conduct the interviews either online, via telephone, or face-to-face. During the telephone-interviews, the participants were at home and the interviewer (AMK) in his office. For the online-interviews we used the data-protected software *Webex* (Version 43, Cisco Systems). The participants were at home and had a camera and microphone to talk to the interviewer, who was in his home office. For the face-to-face interviews we offered interested participants to conduct them in an office at the facilities of the University Medical Centre Hamburg-Eppendorf. Nobody else was present during the interviews. The interviews were held in German. Quotes in this paper were translated into English by the authors.

### Data collection

Data for this study were collected using a semi-structured interview guide, which had been developed during April and May 2023 based on a previous study [29]. While no pwMS were involved in the design of this study, we based our interview guide on an Australian study [29], where they co-designed their interview guide with pwMS as well as MS clinicians. Our initial draft encompassed seven main questions and was discussed within the research team. Feedback from this internal review was further discussed with DK and CHM and two further external experts with experience in the field of smoking/addiction and in qualitative research. This process resulted in a revised version of the interview guide comprising eight main questions (one additional question and some reformulations) complemented by a few optional questions and prompts. The full interview guide has been provided in the supplementary materials.

Prior to the interviews, participants received detailed information about the study design and goals. Participants provided written informed consent and filled out a short questionnaire about demographics (age, sex, employment status, family status, smoking status) and disease-specific characteristics (time of diagnosis, type of MS, PDDS). Informed consent and baseline information were collected online via *LimeSurvey*, a data-protected survey tool provided by the University of Hamburg. PwMS were eligible to participate if they had a self-reported diagnosis of MS of any type, and if they were current smokers, or if they had stopped smoking within the last two years but after their MS-diagnosis. We conducted all interviews between May and July 2023. All interviews were audio-recorded and then transcribed verbatim with the software *f4x* (by dr. dressing & pehl GmbH). AMK then revised and corrected the generated transcripts manually. A copy of the transcripts was sent back to the participants for any comments or corrections. However, none of the participants provided any feedback or requested revision. The analysis of the interviews started concurrently to the conduct of the remaining interviews. We stopped the data collection once AMK and KRL agreed that data saturation was reached, as no new themes arrived from the analysis of the interviews.

### Theoretical framework

The analysis of the data in this paper is based on a realist approach [34, 35]. This approach is often used to better understand and describe the nature of experiences and motivations of people [34]. It is a well-fitting theoretical framework, as this research aims to investigate the motivators and barriers of pwMS regarding smoking cessation as well as their experiences with MS clinicians on this topic. Within this epistemology, asemantic thematic analysis was carried out. This method enables a detailed exploration and description of the collected data, whilst looking for *themes* which represent a “patterned response or meaning within the data set” [34]. A mix of an inductive and deductive approach was chosen. We derived our leading questions of the interview guide from our research questions, therefore deductively influencing the synthesis of our main themes. The sub-themes, however, were derived inductively during coding, as our openly formulated questions allowed our participants to freely mention whatever they thought to be important. This aligned well with our intention to depict and describe a broad spectrum of (sub-)themes and relevant factors to our topic, while also keeping the focus on the main aim of this study. In the same regard, we chose to stay on a semantic level for the analysis. This means, that we focused on the *descriptions* of motivations, barriers and needs of the participants, rather than trying to read “between the lines” and losing the focus on the broader picture too much [34].

### Data analysis

We used the software MAXQDA (Version 2022) for data analysis. To ensure flexibility throughout the process, we followed the six phases for a thematic analysis, as suggested by Braun and Clarke. In summary, this process is about the familiarization with the data, generating codes, searching for themes, and then reviewing, defining and naming them, before writing down the results [34]. In the following, we describe the whole process in more detail:

In the first phase the coders AMK, KRL and BvG familiarized themselves with the data, by reading the transcripts several times (phase 1). The main coder (AMK) started going systematically through the first three interviews, generating first initial codes. By using the inductive approach, the codes did not have to have a direct connection to the research question. This phase generated a set of 34 codes. The set was used by KRL to analyse the data, creating new codes or commenting on given ones if deemed necessary. AMK and KRL then discussed each code on a semantic level, condensed similar codes into one or split codes that needed to be more detailed. This process resulted in the formation and definition of 48 codes in total (phase 2). This final set of codes was then applied to all the other interviews by AMK, KRL and BvG. In the case that the need for a new code arose, the steps of phase two were repeated. After coding all interviews, we looked at which codes fit well together in terms of content, in order to create thematically fitting sub-themes. With this process we organized the 48 codes into seventeen sub-themes (phase 3). The coders used the sub-themes to go back into the data and checked them for coherence within the code- and data-structure. Discussion within the team of coders was used to solve any discrepancies and differences (phase 4). Phases 3 and 4 were repeated until the coders agreed on a clear definition of five main themes, which were based on the seventeen sub-themes and which capture the essence of the data (phase 5). In the Results section the themes and findings are reported in detail (phase 6).

### Reporting of findings

Each quote presented in the findings includes the participant pseudonym (e.g. SC001), the smoking status (S = Smoker, XS = Ex-Smoker), PDDS-category (0–2 = mild, 3–6 = moderate, 7–9 = severe), and time since diagnosis of the respective interviewee (e.g. *SC040*,* S*,* moderate*,* 7 years*). Further, to simplify the terms used to describe the proportions of participants reporting on certain topics, we defined the following categories: A few = less than 25% of participants, some = 26–39%, half = 40–60%, many = 61–75%, most > 75%, as has been applied before [36, 37].

### Ethics

The German ethics committee of the Hamburg Chamber of Physicians approved this research project (registration number 2022–100779-BO-ff).

## Results

### Participants characteristics

The interviews lasted between 15 and 50 min with a mean duration of 30 min. Eight interviews were conducted via telephone, six online and one face-to-face. All detailed participant characteristics can be found in Table 1.

**Table 1:** Sample characteristics (*N* = 15)

Variable	*N* (%) or M (range)
**Sex**	
Female	8 (53.3%)
Male	7 (46.7%)
**Median Age (Range**,** in years)**	47 (27–68)
**Smoking Status**	
Current	11 (73.3%)
Former	4 (26.7%)
**Type of MS**	
Relapsing-Remitting	7 (46.7%)
Primary Progressive	6 (40.1%)
Secondary Progressive	1 (6.6%)
Unsure	1 (6.6%)
**Median Time since Diagnosis in years (Range)**	4 (1–26)
**Median PDDS-Score (Range)**	2 (0–7)
**Employment Status**	
Employed	10 (66.7%)
In Training	1 (6.6%)
Retired	4 (26.7%)
**Family status**	
Single	5 (33.3%)
Married	8 (53.3%)
Separated	2 (23.3%)

*M *Median, *PDDS *Patient Determined Disease Steps

### Themes

During the thematic analysis, we defined our five primary themes as follows: 1) Motivational factors promoting smoking cessation, 2) Perceived barriers preventing smoking cessation, 3) Lack of knowledge about smoking and MS, 4) Unsatisfactory communication with MS clinicians and 5) Identified needs for smoking cessation support.

### Motivational factors promoting smoking cessation

The first theme comprises all motivational factors which promote the decision to quit smoking. While most participants were still current smokers dealing with the decision whether and how to quit, we also had four ex-smokers in our sample, who could tell us about their motivational factors which helped them to quit smoking. Exemplary quotes for this theme and its sub-themes can be found in Table 2.

#### MS

An important aspect for half of our participants, no matter if current- or ex-smoker, were the MS diagnosis and MS-related consequences of smoking. The diagnosis was often described as a very imprinting life event, triggering all sorts of thoughts regarding one’s health behaviour, including the idea to quit smoking. Even when the symptoms of the disease at onset are often only moderate, the apprehension of more severe symptoms of MS is described as a frightening experience by a few participants. Half of the participants also mentioned to have worse symptoms when they smoke, like the feeling that the nerve pathways are “slower”, increased tingling sensations or problems with dizziness and balance. Here, the desire to avoid further uncertainties concerning the prognosis of the disease is explicitly mentioned as a driving motivational factor to at least contemplate smoking cessation.

#### General health

Half of our participants also mentioned their general health, apart from their MS. They listed various health-related disadvantages that come with smoking, like lower physical performance, high blood pressure or nasal congestions. These physical drawbacks also served a motivational purpose towards smoking cessation, as they were hoping for health improvements when quitting smoking.

#### Social factors

Another important aspect were social factors, which were described in multifaceted variations. A few participants, for instance, reported about negative examples of the effects of smoking which stuck in their memory in such a strong way, that it positively fostered their decision to quit. For example: One participant saw a MS-patient in a wheelchair smoking in front of a medical facility. This instituted a negative picture of “what could happen if I continue smoking” in their head, ultimately supporting their decision to quit.

Next to the more repelling pictures influencing the motivation for smoking cessation were also reports of more positive social influences impacting ones’ decision to quit. For example, a few participants explained the positive way their social environment would react, if they were to quit smoking. Related to a positive social resonance is the idea of being a role-model. This factor played a significant role for all of the participants, who recently became parents or had a pregnant partner. The idea of becoming a parent and therefore carrying new responsibilities for a newborn was described as a strong motivator to quit smoking.

#### Money and smell

We want to point out that many participants mentioned at least two more aspects in regards of perceived disadvantages of smoking: the unpleasant smell and the high costs of tobacco. However, in contrast to the other aspects mentioned within this theme and while acknowledging them as disadvantages, none of the participants described the financial and odorous burdens of smoking as motivational factors to at least contemplate smoking cessation.

**Table 2:** Quotes for the theme “motivational factors promoting smoking cessation”

Quote	Sub-Theme
“No, I got my diagnosis three years ago, and I thought about whether it all still makes sense and what I can do to feel better. Then there was also the idea of ​​quitting smoking.” [SC029, XS, mild, 4 years]	MS
“ Yes, definitely the nerves. Because there was a time when I started [smoking] again, and I noticed that when I smoke, my nerves are already impaired right afterward.” [SC023, S, mild, 16 years]	MS
“And besides, yes, I would generally like to quit in the long run for the sake of my body.” [SC041, S, mild, 12 years]	General health
“Now I have to say, my circle of friends and acquaintances is quite extensive, quite large, but it [smoking] has noticeably decreased, and so I also smoke less. I notice that my smoking behaviour adjusts in such a way that you can no longer smoke everywhere. For instance, in restaurants, it has not been allowed for years. And if people around me don’t smoke, I notice in my behaviour that I smoke less too.” [SC025, S, moderate, 4 years]	Social Environment
“For example, I know that my father already had a strong smoker’s cough when I was a toddler, and that’s 20, 30 years ago now. That was always my negative example. I didn’t want to end up like that. So, I always tried to… to separate myself from tobacco because I always had and still have negative connotations with tobacco.” [SC035, XS, mild, 4 years]	Social Environment
“Well, if. If I were to quit smoking now, it would only bring positive feedback from my environment, that is, feedback. They would probably always roll out the red carpet. I don’t know. They would think that’s great.” [SC038, S, severe, 3 years]	Social Environment
“And actually, the main reason was that my girlfriend got pregnant last year, and from that point on, we completely stopped smoking, even party cigarettes.” [SC022, XS, mild, 5 years]	Social Environment
“I just notice that, for example, people who used to smoke, smell incredibly disgusting from their mouths. […] so I will probably smell like that too.” [SC032, S, mild, 4 years]	Money and Smell
“It’s damn expensive. I mean, really a lot of money that you then smoke away.” [SC022, XS, mild, 5 years]	Money and Smell

### Perceived barriers preventing smoking cessation

On the opposite site of motivational factors who drive you towards smoking cessation are the perceived barriers preventing you from becoming active or successful with that intent. Exemplary quotes for this theme and its sub-themes can be found in Table 3.

#### Addiction and habit

A great barrier, which was mentioned by many participants, was their tobacco addiction. They explained that their urge to smoke was most of the time too strong and could not be replaced by another behaviour. This explanation often went hand in hand with the description of withdrawal symptoms, making it very hard to quit smoking.

In the context of addiction and dependency, the participants mentioned not only the physical struggles, but highlighted the significant psychological dependence on smoking. Here, tobacco smoking was described as an integral and habitual behaviour or ritual that often comes in connection with a different behaviour. For example, a cigarette together with a cup of coffee in the morning to start the day, or the cigarette during/after work, for a small break in between more stressful moments. In these cases, the smoking of a cigarette was described by many participants as a little but important “me-time” that mentally allowed them to break out of the every-day-life and to have time for themselves. The psychological aspect becomes especially clear for those participants, that persistently struggled with the idea of quitting over a longer period of time, and who had failed quit attempts in the past. Here, the thought of a withdrawal could cause panic and fear, ultimately preventing the participant from feeling able to quit.

#### Smoking as a coping mechanism

For some participants, smoking served as a coping mechanism, to deal with stressful every-day or life-events. Participants explained that smoking helped them deal with difficult situations like a divorce or stress at work or with family and friends, because smoking a cigarette would help to calm down and escape from those difficulties.

Ironically, one participant cited the MS diagnosis as the primary factor leading them to *resume* smoking. They explained, that when faced with the diagnosis they turned to smoking as a mechanism to cope with their challenging situation.

#### Worries about negative consequences of quitting

When asked for their perceived benefits of smoking, many participants talked about different social aspects. Almost all agreed, that it is very easy to connect with other smokers, may that be at a party with friends and strangers or at work with colleagues. Consequently, when faced with the thought of quitting smoking, a few participants also mentioned the fear of missing out as a barrier for smoking cessation. Some elaborate, that as a non-smoker it would be harder to make social connections and that they would possibly miss out on social opportunities.

Another worry, which was mentioned by a few participants, was a possible change in their mood and resilience. Based on the experience of previous quit attempts, they explained that their mood deteriorates, when they cannot smoke. They said that this is not only an unpleasant experience for themselves, but also for their social surroundings who are possible recipients of their bad mood. One of them further elaborated, that they also feel less resilient towards daily challenges, for instance in the context of work, when they cannot resort to smoking a cigarette. This consequently posed a barrier for them to successfully quit.

Weight gain posed another barrier that a few participants explained in connection with negative experiences from previous quit attempts. On one hand, they mentioned the fear of gaining weight, consequently being unhappy with their body. But on the other hand, they also talked about the negative response they received from their social environment, when they quit smoking and gained weight in the process. This led them to start smoking again in order to shed the extra weight.

One participant explained to us that he tried to quit multiple times, and that after two separate attempts, shortly after quitting, a relapse occurred. While this particular participant still managed to quit smoking eventually, it still highlights that the worry about a relapse induced by withdrawal from tobacco poses a relevant barrier that can prevent smoking cessation or at least make it significantly more difficult.

#### Lack of perceived negative consequences of smoking

While half of our participants reported negative effects of smoking on their MS, a few also reported that they don’t feel any negative impacts or consequences when they smoke compared to when they had stopped for a while. This ultimately posed a barrier towards smoking cessation for them, as they couldn’t identify any MS-related disadvantages for themselves, therefore reducing their motivation to quit.

**Table 3:** Quotes for the theme “perceived barriers preventing smoking cessation”

Quote	Sub-Theme
“However, it’s also an addiction, you know… I can’t just easily say, okay, instead of that, I’ll have a glass of water.” [SC019, S, mild, 2 years]	Addiction and habit
“The psychological dependence is really enormous. You have rituals that allow you to break out of the routine of everyday life or work situations or even stressful situations.” [SC022, XS, mild, 5 years]	Addiction and habit
“Yeah, it’s been like this for years. I think about quitting every day. And yeah, I already get sheer panic at the thought of it.” [SC040, S, moderate, 9 years]	Addiction and habit
“I’ve just gone through a divorce from my husband after 30 years of marriage, which wasn’t easy, especially leaving the security behind. Then, I’m partially disabled because of my MS, but it’s been many years. I still work also… and now I have a really terrible boss and I’m being bullied at work. Really mean… And it stresses me out a lot. And then I notice that as soon as I leave work, I can smoke three cigarettes in a row because I’m so nervous. So, yeah, I think that’s the main reason why I still smoke.” [SC036, S, mild, 15 years]	Coping Mechanism
“For me, it was quite clear that it’s MS, and that’s when I started smoking again because I said to myself, ‘Hey [Name], you have different problems now than not smoking. You have problems with MS.’ And then I started smoking again right there in the hospital.” [SC037, S, severe, 26 years]	Coping Mechanism
“Yeah, why does a smoker smoke? Because they enjoy pulling on a cigarette in the company of other smokers, creating a certain coziness.” [SC025, S, moderate, 5 years]	Worries about negative consequences of quitting
“[…] suddenly I feel like the things I usually enjoy, I don’t enjoy them as much anymore. That’s the first point. And the second point is that I feel like I’m not as resilient anymore. And a big part of my… my self-image and my career path depends on being resilient.” [SC039, S, mild, 4 years]	Worries about negative consequences of quitting
“Because everyone was saying, ‘I’ve gained so much weight.’ Not a little. Quite a bit. And that annoyed me afterwards. At first, I didn’t care. But I think, in general, everyone should look out for themselves rather than how others look. And then the pressure became so great that, yeah, if I smoke, then of course I eat less. Exactly.” [SC023, S, mild, 16 years]	Worries about negative consequences of quitting
“My two relapses oddly occurred after quitting tobacco, so I somehow saw a connection there […] Of course, it could also be a coincidence, but it was indeed peculiar that both times, shortly after quitting, a relapse occurred again.” [SC035, XS, mild, 4 years]	Worries about negative consequences of quitting
“I must say, however, that I really enjoy smoking. Always knowing that it’s anything but healthy. However, regarding my illness, MS, I can’t immediately see the added value of quitting smoking right now.” [SC025, S, moderate, 4 years]	Lack of perceived negative consequences of quitting

### Lack of knowledge about smoking and MS

We asked pwMS to tell us what they know about the connection between tobacco smoking and MS and where they get their information from. Exemplary quotes for this theme and its sub-themes can be found in Table 4.

#### Knowledge

The level of knowledge among participants was low. Many *assumed* a negative connection between smoking and disease progression, but often were not sure about it. Even if they expressed reliance on information they had read or heard, they often remained uncertain about its accuracy. Only a few confidently stated the negative effects smoking can have on the disease progression or onset. The uncertainty about specific knowledge on smoking and MS was often emphasized by the use of many indeterminates like “*I think”*, “*maybe*” or *“could be”.*

In a few other cases, the (perceived) knowledge was based only on personal experience or from family, friends or acquaintances experiences, were given great importance by the participants. They used these to conclude possible impacts that smoking can have on their MS activity and progression (see Table 4).

#### Source of information

We also asked specifically about the participants’ sources of information they used or would use, regarding the connection between smoking and MS. Here, the website of the German Society for MS was commonly mentioned. A few participants mentioned that they would “have a look on the internet”, without naming specific websites or organizations. A few other participants, on the other hand, said that they would ask and talk to their neurologist or general practitioner for more information about the topic or ask them for specific material, like brochures.

**Table 4:** Quotes for the theme “lack of knowledge about smoking and MS”

Quote	Sub-Theme
“Um, well, MS is a nerve thing. And I don’t know if smoking triggers the nerves. I don’t know because… Ah, I don’t know.” [SC038, S, severe, 3 years]	Knowledge
“I have the feeling that smoking could have caused my relapses.” [SC035, XS, mild, 4 years]	Knowledge
“I also have another acquaintance who… she was also a heavy smoker and also has MS, and I believe she has a similar situation. So, you gather information from all sorts of places, like friends, if you also have acquaintances who have it, then you naturally have a whole different, a whole different well of information.” [SC035, XS, mild, 4 years]	Source of information
“Yeah, as I said, my doctor, of course. And then the internet, naturally. Otherwise, I wouldn’t know where to find information about it.” [SC036, S, mild, 15 years]	Source of information

### Unsatisfactory communication with MS clinicians

The analysis of participants’ experiences with MS clinicians revealed a consistent theme of unsatisfactory communication regarding smoking and MS and a perceived misconception from the clinicians’ side. Exemplary quotes for this theme and its sub-themes can be found in Table 5.

#### Lack of assistance

Main points mentioned by most participants were a lack of guidance, only brief mentions of the topic without follow-up support, a general perceived lack of support as well as minimal practical assistance and misconceptions from the clinician’s side.

Most participants reported that a proper conversation on the topic smoking and MS simply did not happen. When smoking was addressed by clinicians, the general advice to quit was common, but practical assistance, specific education on the topic, and guidance were lacking. A few were even frustrated by hearing the common advice “you should quit smoking”, without receiving any concrete support or being referred to professional smoking cessation support.

#### Perceived misconception

These unsatisfactory experiences were perceived by a few participants as a misconception of the problem on the clinicians’ side. They told about their experience, that clinicians thought that the ability to quit is solely dependent on one’s willpower and that pwMS who still smoke simply don’t want to quit, leaving the participants without further guidance or support.

**Table 5:** Quotes for the theme “unsatisfactory communication with MS clinicians”

Quote	Sub-Theme
“No, there wasn’t really any conversation about it, and nothing was recommended. So, I didn’t get any advice on how to quit smoking at any point.” [SC019, S, mild, 2 years]	Lack of assistance
“But yeah, like I said, specifically regarding MS, I really haven’t had any significant conversations. Unfortunately.” [SC035, XS, mild, 5 years]	Lack of assistance
“In the last conversation I had with a doctor, it was like, ‘Yeah, it would be good if you could quit,’ but that was it. It’s always just briefly mentioned, and then no one says where to go, where to get help, where it gets better, what you could do. It’s always just briefly touched upon.” [SC029, XS, mild, 4 years]	Lack of assistance
“Everyone says, of course, quit, but practically no assistance. … The opinion always circulates that you just have to want to. And if I don’t quit, then I don’t want to. … Yes, that’s it.” [SC040, S, moderate, 9 years].	Perceived misconception

### Identified needs for smoking cessation support

The needs and wishes for support and assistance of pwMS were often based on perceived barriers preventing smoking cessation and their experiences from previous quit attempts. Exemplary quotes for this theme and its sub-themes can be found in Table 6.

#### Informative conversations with MS clinicians

When asked, what kind of assistance they would like to receive in the future for successful smoking cessation, participants’ statements revealed a shared recognition of the need for more comprehensive support and guidance in the process. Half of them expressed a desire for a simple but more specific and informative conversation with experts, by highlighting the importance of a dedicated discussion that explains the specific effects of smoking on MS.

Also, half of our participants further mentioned that they would prefer to have the conversation about smoking cessation with their neurologist, rather than with their general practitioner or any other expert. Here, they hoped to receive the best information regarding smoking and MS. Getting specific information from a neurologist would put extra weight on the importance of smoking cessation regarding their MS, therefore increasing their motivation to quit.

#### Smoking cessation course

Some, on the other hand, wished for and specifically considered smoking cessation programs outside of a clinical setting. Yet, explanations of the participants revealed, that they want those programs to be expert-led with an educational focus on health-related topics regarding smoking and MS. Within these programs, they wished for easy-to-understand numbers or figures, which showcase the most important data from relevant studies dealing with smoking cessation in pwMS. When asked for the preferred mode of delivery of a potential smoking cessation program, answers from the participants did not reveal a clear preference towards an online or face-to-face course. A few participants highlighted the advantages of an online course, for instance the comfort of being able to stay at home. Yet others explained, that they prefer to speak to others face-to-face and were willing to travel to a specific location for this purpose.

#### Support in the home-setting

A few participants who had negative experiences with trying to quit in the past were specifically calling for more support in the home-setting. Here, they emphasized the importance of a longer-term support within the familiar home environment. They believed that sustained support in one’s habitual surroundings would be more beneficial for the cessation journey, as previous quit attempts - which for example starting during a rehabilitation measure in a clinical setting - failed as soon as they got back into their home-setting.

#### Peer-exchange

Peer-exchange was mentioned by few participants as a beneficial factor that would support them to quit smoking. They emphasized the value of sharing positive experiences and providing encouragement from other people with MS to make the process seem more achievable and relatable, as this approach aims to spread the idea that quitting is possible and not as difficult as one might think.

#### Nicotine replacement therapy (NRT)

When asked if they ever tried any kind of NRTs, like nicotine gum, most participants said that they either never tried them or disliked them. Only one participant described them as a helpful tool for smoking less. Those who never tried NRTs were very sceptical about them, saying that it would not feel “right” or make sense to use them, as they still contain the addictive nicotine, therefore not tackling the root of the problem.

**Table 6:** Quotes for the theme “identified needs for smoking cessation support”

Quote	Topic
“But I really wished for a conversation. Someone who really explained to me for about ten minutes what smoking triggers and explained it a bit more specifically.” [SC016, XS, mild, 3 years]	Informative conversations with MS clinicians
“I think maybe even a neurologist… who might explain it a bit more concretely in terms of the effects on my MS… Those are the… areas where I think it could be most beneficial for me.” [SC041, S, mild, 12 years]	Informative conversations with MS clinicians
“I could imagine that even a smoking cessation course is probably the most effective because you also get information from the expert on how the body reacts to such things and what damage is done by smoking and what dangers are involved.” [SC035, XS, mild, 4 years]	Smoking cessation course
“I think both would be good. So, it would be private online, too, as there would be fewer people… I mean, in group therapy, when there are 20 people sitting there, you probably feel like, well, they don’t even hear me up there, but I think face-to-face would be good regardless of whether it’s online or now… really sitting across from each other and discussing it.” [SC029, XS, mild, 4 years]	Smoking cessation course
“There are so many different approaches. But I think a longer, more sustained support in the home environment, in the familiar surroundings, is important. That would make a difference.” [SC037, S, severe, 26 years]	Support in the home setting
“ ’Hey, look, it’s super easy. I quit from one day to the next. Come on, let’s not smoke today, even if we have a beer’. So, passing on this experience, trying to spread the idea that it’s possible and that it’s easy. I think that might have been a good way, at least for me.” [SC022, XS, mild, 5 years]	Peer exchange
“I once tried those weird nicotine gums, but I found them awful. No, I haven’t tried anything else yet. I also don’t know what’s currently available. I’ve thought about those electronic cigarettes before, but in principle, they’re just another form of cigarettes.” [SC036, S, mild, 15 years]	Nicotine replacement therapy

## Discussion

**Fig. 1:**
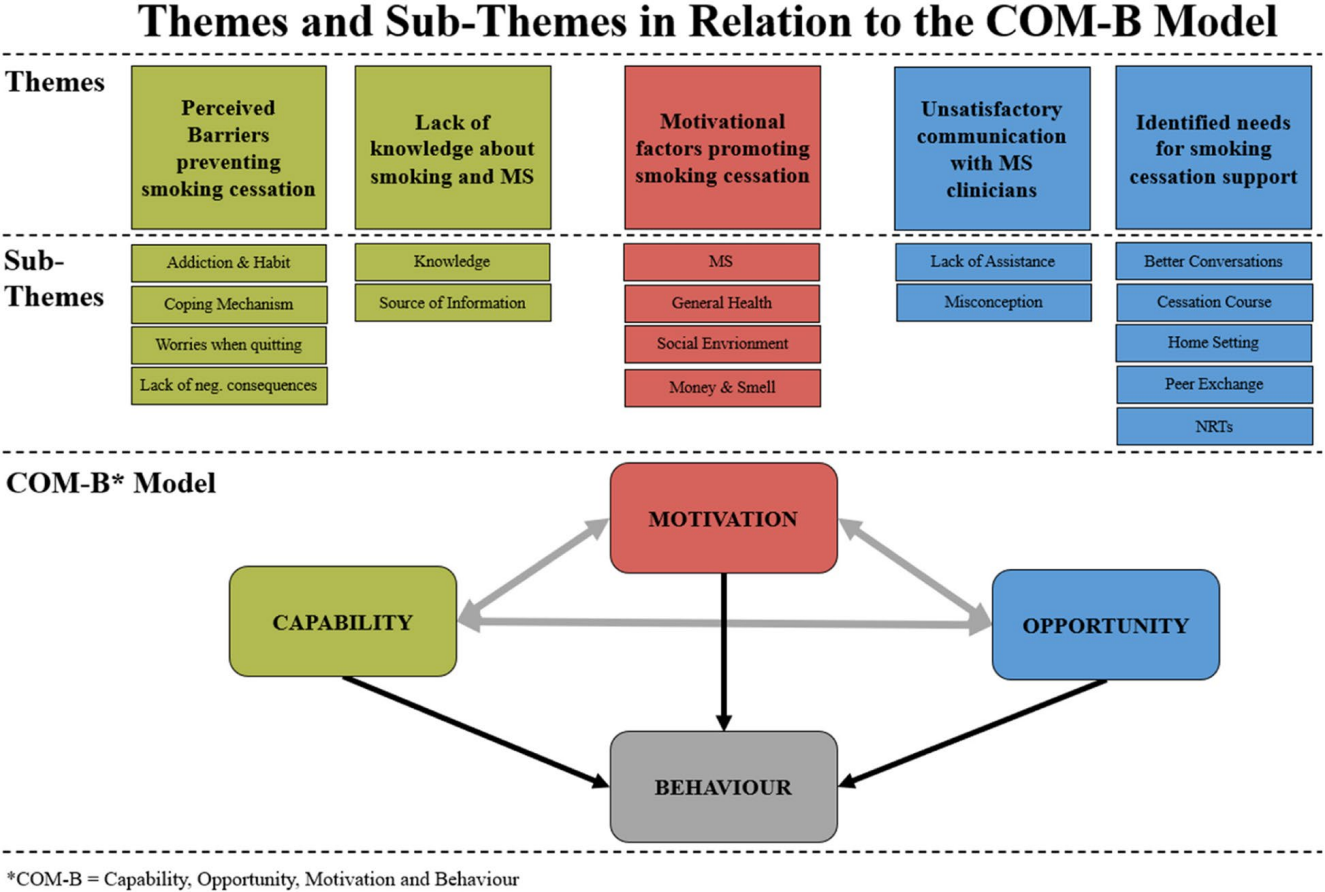
Themes and sub-themes in relation to the COM-B model (*COM-B = Capability, Opportunity, Motivation and Behaviour)

This study is the first to our knowledge to investigate the motivators, barriers, needs and wishes of pwMS regarding smoking cessation in Germany, where smoking prevalence is particularly high (28,2%) [22]. As mentioned in the introduction, this study suggests the COM-B model as a behavioural framework to give first indications about what needs to change for pwMS in order to inform the development of a MS-specific smoking cessation intervention. In this section we categorize and discuss our findings as “general” and “MS-specific” findings and are mapping them onto the COM-B model. As a reminder: the model uses *Capability*,* Opportunity* and *Motivation* to explain a (new) *Behaviour* [31]. Figure 1 gives an overview of our themes, sub-themes and their respective place within the model.

### General findings

Some identified motivators within our theme “Motivational factors promoting smoking cessation” were similar to findings in the general population. A study from Halpern and Warner [38] identified health-related concerns and pregnancy as most relevant motivational factors for smoking cessation among 7700 current and ex-smokers, validated by more recent studies [39–41]. Other frequently mentioned aspects by our participants like financial considerations or the smell of tobacco smoke were perceived as disadvantages, but did not contribute to the motivation to quit. These findings are also supported by other findings [38], where concerns about the costs of cigarettes even contributed to a decreased likelihood of quitting. However, these results are not unchallenged, as other studies indeed identified the costs of cigarettes and the unpleasant smell of tobacco as effective motivators for smoking cessation [39, 41, 42]. Within the COM-B model, these points can influence the *Motivation* of a person, as they might feel more motivated to perform a new *Behaviour*, if they think, for example, that it will positively influence their general health.

One frequently mentioned aspect within our theme “Perceived barriers preventing smoking cessation” was tobacco addiction. It is unsurprising that physical addiction poses a barrier to quit [41] with the level of nicotine dependence being closely related to quitting success [43, 44]. In this context, the psychological dependence on smoking has also been emphasized by our participants, where smoking serves as a coping mechanism to navigate everyday stress or perceived changes in mood and resilience. The National Young Adult Health Survey from the United States found in this regard, that the “loss of a way to handle stress” for many facilitates a great barrier for smoking cessation [42], which stands in line with other findings which show that “tension reduction” is a motivator for continued smoking [45].

Another aspect within the same theme brought up by a few participants was weight gain as a result of smoking cessation. This worry is supported by findings, that if people gained weight after quitting, they were more likely to relapse and pick up smoking again [46], therefore facilitating weight gain as a relevant barrier for smoking cessation. In regard to the COM-B model, both addiction and worry about negative consequences like weight gain, are influencing the *Capability* of a person, as they might feel less capable of performing a new *Behaviour*, if e.g. addictive behaviours are standing in their way.

### MS-specific findings

Within the theme “Motivational factors promoting smoking cessation”, we could identify MS-related health concerns as a relevant motivator in our sample. Half of our participants mentioned at least some kind of negative impact that smoking has on their MS. Negative effects on MS symptoms were also identified in an Australian study as a motivator to quit smoking [47]. Showcasing how symptoms might get better after smoking cessation could therefore be an efficient way to increase motivation among pwMS. This approach should, however, take into account the worry that withdrawal symptoms during cessation might negatively impact MS symptoms and relapses, as mentioned by one of our participants, and by said study [47]. However, there are no studies indicating that quitting smoking causes relapses in pwMS. Here, the small risk of temporarily increasing stress causing a relapse [48] is thought to be outweighed by the benefits of quitting on the long-term health and wellbeing of pwMS. Similar to the findings about general health concerns and in regard to the COM-B model, the MS-related health concerns can influence the *Motivation* of a person to perform a new *Behaviour*.

The findings within our theme about “Lack of knowledge about smoking and MS”” underscore the need for clearer dissemination of information and education about the topic. Most participants were unsure or unaware of the negative connection between smoking and MS, which is in line with the mentioned Australian study [47]. Participants knew very well that smoking was not good for their general health but they lacked knowledge and awareness about the negative consequences of smoking on MS progression and had misconceptions about the use of NRTs. Within the COM-B model, knowledge can be seen as a resource, which, if missing, influences the *Capability* of a person to perform a new *Behaviour.*

In our theme about “Unsatisfactory communication with clinicians” our participants emphasized, that MS-specific education about the topic and recommendations for smoking cessation support or guidance were lacking. Superficial advice, simply telling the patients that smoking is bad and that they should stop, was rated as not helpful and even perceived as frustrating. This points at a missed opportunity, as behavioural interventions like specific advice from clinicians can increase smoking cessation rates [49–51], and additional evidence shows that quit rates can be improved if clinicians use referral pathways for further assistance [52]. Our results are in line with other studies outside of Germany that show that only 4 − 15% of (ex-)smokers received help from a clinician with smoking cessation [41, 53]. Some of our participants also emphasized that they would value the advice even more coming from a MS clinician, rather than a general practitioner, as they are perceived to be more qualified to talk about the MS-specific aspects of smoking. These findings stand in line with the results of an Australian online survey among pwMS [54].

Further, within the theme “Identified needs for smoking cessation support”, many of our participants demanded expert-led programs, and longer-term support, also in the home setting, as well as peer-exchange. In the above-mentioned Australian survey study [54], more than half of the 69 smoking participants also expressed interest in a smoking cessation program especially tailored for pwMS. This fits with the descriptions of our participants, where some explained that they would value the idea of participating in such a program, may that be online or face-to-face. Further, they expressed the wish for peer-exchange. A systematic review analysing the effectiveness of different smoking cessation interventions among adults found evidence, that tailored self-help groups can significantly contribute to successful smoking cessation [55]. Addressing and enabling peer-exchange among pwMS could therefore be of high importance in developing more effective smoking cessation strategies.

When translating the findings of the themes “Unsatisfactory communication with clinicians” and “Identified needs for smoking support” onto the COM-B model, we find that they both influence the *Opportunity* of a person. PwMS might have better opportunities to change their smoking *Behaviour*, if they have, for example, a satisfactory counseling with an MS-clinician about smoking, and join a MS-specific and expert-led cessation program, compared to someone, who doesn’t get these opportunities.

Given that some of the motivators and barriers of pwMS are similar to the general population, it will be a useful step to base a MS-tailored intervention on an already existing generic smoking cessation program. The tailoring of such a program should then focus on the integration of the identified aspects that are unique to pwMS. For example: an online, expert-led, group-based smoking cessation program might be a feasible way to transfer information about the connection of smoking and MS to improve knowledge and motivation. Ensuring that MS symptoms, which may hinder cessation, are managed appropriately may also increase smoking cessation success. Also, the participants of the program would get the chance to connect with and provide peer-support for each other, which would further account for the findings of the theme about identified needs. Similar methodological approaches have been used in other generic smoking cessation programs. However, adherence, acceptability and feasibility for an MS-specific program will need thorough testing. The identified unsatisfactory communication with MS clinicians, on the other hand, has potential implications for the current practice in the clinical setting. Further research should investigate the healthcare provider’s views on the topic and analyse what support they need to provide pwMS with better information and clinical assistance for smoking cessation, since MS-clinicians could also play a vital role in referring pwMS to MS-specific smoking cessation interventions.

### Strengths and limitations

Most participants were recruited via our own facility, because our newsletter seems to have reached possible participants more efficiently than our advertisements on the DMSG-website or at our partnered facility. While we cannot completely rule out a selection bias that might have influence on the participants knowledge and their experiences they might have had with quit attempts, we don’t expect any significant differences in these matters. Most of our participants’ characteristics are well distributed, but we included mostly people with mild or moderate health restrictions due to their MS. Marck et al. [28] argue, that pwMS with more acute MS-related problems and symptoms (e.g. pain, depression, or cognitive dysfunction) might report motivators and barriers concerning smoking cessation that are more unique to this patient group. Hence, we cannot rule out that we could have found more factors influencing smoking cessation that are unique to pwMS in a sample with a higher average PDDS-score. Further, we only documented self-reported MS. However, we expect the risk of false self-report in this matter to be low, since most participants were patients at our MS day-clinic.

By including both, current and ex-smokers in our sample, we broadened the spectrum of different perspectives on the topic of smoking cessation. Especially ex-smokers have a unique insight on the difference between previously failed and their ultimately successful quit attempt. The inclusion of these participants helped us to gain a better understanding on what is important to successfully quit. Also, by offering every participants to conduct the interview either online (with camera), via telephone or face-to-face, everyone could adapt for an interview-situation they felt most comfortable with. This can be important to avoid bias when discussing sensible topics like health concerns or addiction.

## Conclusion

While some of our identified aspects are similar in pwMS compared to the general population, like addiction and health-related concerns, we also identified MS-specific factors influencing smoking cessation, most importantly the lack of knowledge about the negative impact of smoking on MS onset, progress, and symptoms. The translation of these findings onto the COM-B model can serve as a first important step towards designing a tailored smoking cessation intervention for pwMS.

## Supplementary Information

Supplementary Material 1.

## Data Availability

The datasets used and analysed during the current study are available from the corresponding author on reasonable request.

## Abbreviations

COREQ: Consolidated criteria for reporting qualitative studies
DMSG: Deutsche Multiple Sklerose Gesellschaft / German Society for MS
MS: Multiple sclerosis
NRT: Nicotine Replacement Therapy
PDDS: Patient determined disability scale
pwMS: People with multiple sclerosis
