# Supplementary material for: Motivators and barriers for smoking cessation in people with multiple sclerosis: a qualitative study to inform the design of a tailored intervention
Source: BMC Public Health. 2024 Dec 17;24:3402. doi: 10.1186/s12889-024-20998-5 (PMC11653690; doi:10.1186/s12889-024-20998-5)
Supplement: Supplementary file 1 — Supplementary Material 1. [file 12889_2024_20998_MOESM1_ESM.docx]

| Supplementary Table 1: Interview Guide | | |
| --- | --- | --- |
| Leading question | Check (Was it mentioned? If not, ask the following questions) | Follow-Up Questions |
| Part 1: Background and Smoking Behaviour | | |
| Tell me how you started smoking and what role smoking has played in your life since then. | Since when have you been smoking?  How much do you smoke on average per day?  Do your friends and family smoke too?  For ex-smokers:  From when to when did you smoke?  Why did you quit smoking? |  |
| What benefits do you experience / have you experienced through smoking?  What disadvantages do you experience / have you experienced through smoking? | Were (MS-specific) advantages AND disadvantages mentioned?  What reasons do you have for continuing to smoke? | Has your attitude towards smoking changed since the MS diagnosis? Please explain. |
| Current smokers: Have you ever tried quitting? Please tell me about it. If not: why so?  Ex-smokers: Please tell me about your decision to stop smoking and how you managed to accomplish that. | What reasons do you believe led to the failure, from your perspective?  What motivations drove you? (MS diagnosis?)  What experiences have you had?  What support and assistance have you sought?  What obstacles did you encounter?  Have you ever used nicotine replacement products?  Do you use e-cigarettes or heated tobacco products? What are their advantages and disadvantages compared to cigarettes?  Are you exposed to secondhand smoke?  For ex-smokers: Were there also failed attempts to quit? | For smokers: Are you currently considering quitting? What thoughts are going through your mind regarding this? (Check: Do you have any concerns about this?)  For ex-smokers who have had failed attempts: What was different this time? Are you considering starting smoking again? |
| Part 2: Assistance | | |
| What has been your experience with medical advice and support regarding smoking? | When or in what context did you specifically receive medical advice and support?  Was there advice and support at other doctor appointments?  Did you find the medical advice and support helpful? In what ways? What exactly was recommended?  What might have been missing? What would you have liked to be different?  Did the doctor refer you to further support services?  Who exactly did you speak to about this topic: general practitioner, neurologist, other healthcare personnel?  Were MS-specific aspects addressed? | What kind of support would have been helpful for you to quit smoking? (Check: Help with MS symptoms? Online or on-site assistance? Which doctor/expert is best suited to address the issue of smoking?)  If there hasn't been a discussion about smoking: Would you have wished for it or preferred to bring it up yourself? Please explain. |
| Part 3: Knowledge | | |
| In your understanding, how are smoking and MS related? | Information sources? (Internet, doctor's consultation, etc.)  Are you familiar with information about the influence of smoking as a trigger for MS?  Are you familiar with information about the influence of smoking on the course of MS? |  |
| Have you ever tried cannabis? What role does it play for you? | How do you consume cannabis? (Joint, vaporizer, etc.) |  |
| Are there any aspects we haven’t talked about yet that you would like to add? |  |  |
